# Maternal plasma angiogenic and inflammatory factor profiling in foetal Down syndrome

**DOI:** 10.1371/journal.pone.0189762

**Published:** 2017-12-15

**Authors:** Monika Zbucka-Kretowska, Karol Charkiewicz, Joanna Goscik, Slawomir Wolczynski, Piotr Laudanski

**Affiliations:** 1 Department of Reproduction and Gynecological Endocrinology, Medical University of Bialystok, Bialystok, Poland; 2 Department of Perinatology and Obstetrics, Medical University of Bialystok, Bialystok, Poland; 3 Faculty of Computer Science, Bialystok University of Technology, Bialystok, Poland; Medical University Innsbruck, AUSTRIA

## Abstract

**Objective and design:**

Angiogenic factors are proteins that are related to certain foetal chromosomal abnormalities. The aim of this study was to determine the concentration of 60 angiogenic factors in the plasma of women with offspring possessing trisomy 21/Down syndrome (DS).

**Method:**

After analysing karyotyping results, we selected 20 patients with foetuses possessing DS, and for the control group, we selected 28 healthy patients with uncomplicated pregnancies who delivered healthy newborns at term (i.e., 15–18 weeks of gestation). To assess the concentration of proteins in the blood plasma, we used a protein macroarray which enabled simultaneous determination of 60 angiogenic factors per sample.

**Results:**

We observed a statistically significant increase in the concentration of these five angiogenic and inflammatory factors: TGFb1 (p = 0.039), angiostatin (p = 0.0142), I-309 (p = 0.0476), TGFb3 (p = 0.0395), and VEGF-D (p = 0.0173)—compared to concentrations in patients with healthy foetuses.

**Conclusion:**

Our findings suggest that angiogenic factors may play role in DS pathogenesis.

## Introduction

Down syndrome (DS), the most frequent chromosomal aberration, is caused by an extra chromosome 21 or a fragment thereof [1]. Based on estimates from 2006 by the National Institute of Child Health and Human Development, the incidence of DS in the United States is estimated at between 1:800 and 1:1000 live births. DS occurs equally often across ethnic and social groups [2] and is a disease of congenital anomalies, which includes congenital heart defects, gastrointestinal anomalies, immune system defects, thyroid disease, bone defects, genitourinary system defects, strabismus, mental retardation, and many other conditions [3].

A pregnancy involving a DS foetus is accompanied by a great number of biochemical variations in the maternal plasma, likely induced by the additional chromosome 21. Our recent research in reference to other articles, shows that pregnancies with foetal chromosomal aberrations are strongly connected with an imbalance in chemokines and bioactive lipids, such as sphingolipids, which may suggest new potential pathomechanisms in foetal DS [4–8]. Additionally, the status of selected cytokines and angiogenic factors in amniotic fluid from chromosomally abnormal pregnancies has also already been described in our research [9–11].

In the scientific literature scientists in recent studies demonstrates that most of the differentially expressed genes in trisomy 21 play a role in angiogenesis, immune responses and inflammation processes [5]. The clinical observations suggest that individuals with DS have a significantly lower incidence of angiogenesis- dependent diseases, with scientists noting the strong endogenous angiogenesis inhibitors’ activity. Current pharmacological cancer treatment is focused on the inhibition of angiogenic factors (or its receptors). Since individuals with DS present an antiangiogenic state due to increased activity of endogenous angiogenic inhibitors, one can speculate that the endogenous angiogenic inhibitors are potential candidates for future cancer therapy [12]. Moreover, Hattori et al. have found evidence for the hypothesis that disturbed concentrations of some chemokines, angiogenic factors and different circulating metabolites in the blood of pregnant women can stimulate a mother’s immune response to produce auto-antibodies directed against these proteins [13]. Scientists have determined the impact of the reduced number and altered morphology of endothelial progenitor cells on the impaired process of angiogenesis and immune response in individuals with DS [5]. Therefore, measurement of the poorly tested angiogenic and inflammatory factors in pregnancies with foetal chromosomal abnormalities could lead to a better understanding of the influence of DS on such pregnancies.

The results of this research could enable a better understanding of abnormal foetal development processes and could direct future investigations for the modification of the developmental process of foetuses with trisomy 21. Since the pathology of the syndrome is extremely complicated, and the additional chromosome 21 causes myriad foetal pathologies with imbalance of angiogenic factors and they can induce the maternal and foetal immunology system, identifying abnormal signals and understanding the pathological mechanism(s) is highly desirable. Therefore major goal of this research is description of potential role of angiogenic factors in patomechanism of foetal DS and interaction between mother and foetal body.

## Materials and methods

The study and control groups consisted of women who underwent routine amniocentesis between the 15^th^ and 18^th^ week of gestation at the Department of Reproduction and Gynecological Endocrinology of the Medical University of Bialystok, Poland. Recruitment occurred between September 2012 and March 2015. We recruited only non-febrile women without any chronic or acute disease, and we excluded women taking any hormonal or anti-inflammatory treatment, as well as those with vaginal or urinary tract symptoms that would suggest infection.

The study protocol was approved by the Local Ethical Committee of Medical University of Bialystok, Poland, and informed written consent was obtained from each participant (ethics committee approval no: R-I-002/36/2014). We obtained 10 mL of peripheral blood, which was collected for EDTA probes after amniocentesis, from the participants. The blood was then centrifuged, and the plasma was subsequently separated and frozen at −80°C. After analysis of the karyotyping results, we chose 20 women with foetuses with trisomy 21, and for the control group, we selected 28 healthy patients with uncomplicated pregnancies who delivered healthy newborns at term.

To determine the concentration of angiogenic factors in the blood plasma, we used a multiplex method as previously detailed [8,10], which allows for simultaneous determination of 60 proteins per sample. Like a traditional sandwich-based ELISA, this method utilizes a pair of specific protein antibodies for detection. A capture antibody is first bound to the glass surface. After incubation with the sample, the target angiogenic factor is trapped on the solid surface. A second biotin-labelled detection antibody, which can recognize a different isotope of the target factor, is then added. The protein factor-antibody-biotin complex is then visualized through the addition of the streptavidin-labelled Cy3 equivalent dye using a laser scanner (GenePix 4100A) and the following two software programs: GenePix Pro7 and Q-Analyzer [11, 13–15].

The sets (Human Angiogenesis Array 1000, RayBiotech Inc.) consist of the following angiogenic factors: activin A, agouti-related protein (AgRP), angiopoietin 1, angiopoietin 2, angiogenin, angiostatin, angiopoietin-like 4 (ANGPTL4), basic fibroblast growth factor (bFGF), chemokine (C-X-C motif) ligand 16 (CXCL16), epidermal growth factor (EGF), C-X-C motif chemokine 5 (ENA-78), fibroblast growth factor 4 (FGF-4), follistatin, granulocyte-colony stimulating factor (G-CSF), granulocyte-macrophage colony-stimulating factor (GM-CSF), chemokine (C-X-C motif) ligand 1 (GRO), heparin-binding EGF-like growth factor (HB-EGF), hepatocyte growth factor (HGF), chemokine (C-C motif) ligand 1 (I-309), interferon gamma (IFN-gamma), insulin-like growth factor 1 (IGF-1), interleukin 10 (IL-10), interleukin 12 p40 (IL-12 p40), interleukin 12 p70 (IL-12 p70), interleukin 17 (IL-17), interleukin 1-alpha (IL-1 alpha), interleukin 1-beta (IL-1 beta), interleukin 2 (IL-2), interleukin 4 (IL-4), interleukin 6 (IL-6), interleukin 8 (IL-8), C-X-C motif chemokine 10 (IP-10), chemokine (C-X-C motif) ligand 11 (I-TAC), leptin, leukaemia inhibitory factor (LIF), monocyte chemotactic protein 1 (MCP-1), monocyte chemotactic protein 2 (MCP-2), monocyte chemotactic protein 3 (MCP-3), monocyte chemotactic protein 4 (MCP-4), matrix metalloproteinase-1 (MMP-1), matrix metalloproteinase-9 (MMP-9), platelet-derived growth factor BB (PDGF-BB), platelet endothelial cell adhesion molecule (PECAM-1), placental growth factor (PLGF), chemokine (C-C motif) ligand 5 (RANTES), transforming growth factor alpha (TGF alpha), transforming growth factor beta 1 (TGF beta 1), transforming growth factor beta 3 (TGF beta 3), tyrosine kinase with immunoglobulin-like and EGF-like domains 1 (Tie-1), tyrosine kinase with immunoglobulin-like and EGF-like domains 2 (Tie-2), metallopeptidase inhibitor 1 (TIMP-1), metallopeptidase inhibitor 2 (TIMP-2), tumour necrosis factor alpha (TNF alpha), tumour necrosis factor beta (TNF beta), thrombopoietin (TPO), urokinase receptor (uPAR), vascular endothelial growth factor (VEGF), vascular endothelial growth factor 2 (VEGFR2), vascular endothelial growth factor 3 (VEGFR3), and vascular endothelial growth factor D (VEGF-D).

Descriptive statistics including the mean concentration and standard error of the mean were calculated for selected factors, henceforth referred to as “features”. To detect statistically significant differences between considered groups (i.e., DS group versus control group), either analysis of variance or a non-parametric method (e.g., Wilcoxon rank-sum test) was applied. The choice of an appropriate method was made upon fulfilling the normality and homogeneity of variance assumptions, and in the case of violation of at least one condition, a non-parametric approach was employed.

The normality of features distribution was checked with the Shapiro-Wilk test [16] and the homogeneity of variances using Levene’s test [17]. A statistical significance level of p = 0.05 was applied for all statistical tests.

## Results

Clinical characteristics of the patients are presented in Table 1. Patients from both groups were matched for maternal age, number of pregnancies, and gestational age at data collection to ensure that there were no statistically significant differences between the groups. Mean concentrations and standard errors of maternal plasma angiogenic factor concentrations in each study group are presented in Table 2.

**Table 1:** Clinical characteristic of the patients.

	Group I–Pregnancies without Down Syndrome (n = 28)	Group II–Pregnancies with Down Syndrome (n = 20)
Maternal age (median ± SD)	37.5 ± 6.83	36 ± 6.61
Number of pregnancies (median ± SD)	1.5 ± 1.89	2 ± 1.05
Gestational age at data collection point (weeks) (median ± SD)	16 ± 1.05	16 ± 0.89

SD—standard deviation

**Table 2:** Concentrations of angiogenic factors in maternal plasma.

	Group I–Pregnancies without Down syndrome (n = 28)	Group II–Pregnancies with Down syndrome (n = 20)	P-value
	Angiogenic factors concentration (pg/ml) Mean ± SEM		Group I- Group II
Activin-A	1249. 86 ± 426.49	2572.85 ± 1460.47	0.6098
AgRP	551.52 ± 146.31	520.77 ± 92.23	0.767
Angiogenin	6876.47 ± 1290.16	6054.87 ± 1354.91	0.5931
ANG-2	3866.02 ± 338.3	3181.5 ± 294.15	0.2844
ANGPLT4	79867.36 ± 14780.22	103653 ± 34218.56	0.9125
bFGF	489.66 ± 56.1	760.92 ± 124.44	0.0916
ENA-78	4664.2 ± 842.18	2885.99 ± 602.66	0.1685
GRO	363.17 ± 51.75	567.12 ± 101.21	0.0574
HB-EGF	6.55 ± 1.75	40.38 ± 26.73	0.0938
HGF	372.31 ± 38.56	533.04 ± 106.51	0.6314
IFN-gamma	336.55 ± 36.4	385.78 ± 75.24	0.7682
IGF-I	17424.73 ± 2373.29	20000.79 ± 3554.24	0.9601
IL-1a	187.21 ± 26.77	266.44 ± 72.22	0.4404
IL-2	554.63 ± 79.27	713.13 ± 153.98	0.3851
IL-6	162.31 ± 13.28	193.75 ± 26.09	0.7449
IL-8	88.07 ± 8.48	106.54 ± 12.99	0.3068
IL-17	557.65 ± 129.12	800.92 ± 183.13	0.358
IP-10	101.003 ± 68.35	91.9 ± 36.57	0.8008
Leptin	11825.51 ± 1484.38	14814.36 ± 3998.67	0.873
LIF	1260.48 ± 282.37	2567.52 ± 898.91	0.2898
MCP-1	99.47 ± 11.22	141.08 ± 30.24	0.7015
PDGF-BB	425.16 ± 52.21	453.9 ± 96.12	0.671
PIGF	445.49 ± 60.006	598.81 ± 122.5	0.6454
RANTES	3853.96 ± 982.76	6664.86 ± 2213.77	0.2399
TGFb1	9776.61 ± 1268.2	14464.41 ± 2025.33	0.039**
TIMP-1	40833.58 ± 3103.84	33269.67 ± 3052.19	0.0894
TIMP-2	11131.3 ± 834.08	12615.48 ± 1529.79	0.6962
TNF-alpha	471.31 ± 121.27	641.41 ± 188.49	0.1137
TNF-beta	156.1 ± 28.17	274.62 ± 114.01	0.5716
TPO	11662.06 ± 1367.79	14576.39 ± 2867.99	0.6061
ANG-1	401.61 ± 105.25	398.18 ± 100.48	0.9836
Angiostatin	183088.02 ± 8761.9	218713.15 ± 10762.9	0.0142*
CXCL16	569.76 ± 53.1	727.91 ± 89.3	0.1473
EGF	6.77 ± 1.31	12.95 ± 3.44	0.186
FGF-4	3156.65 ± 1160.05	16633.63 ± 8734.45	0.1673
Follistatin	17023.61 ± 2703.59	19053.72 ± 3452.04	0.7814
G-CSF	2766.98 ± 745.4	3648.46 ± 1590.008	0.772
GM-CSF	36.42 ± 12.37	42.94 ± 19.89	1
I-309	25.93 ± 4.05	60.42 ± 15.01	0.0476*
IL-1b	60.97 ± 10.19	93.96 ± 25.29	0.9917
IL-4	46.26 ± 4.02	50.52 ± 6.09	0.5432
IL-10	12.12 ± 2.008	19.71 ± 6.48	0.5848
IL-12p40	587.85 ± 96.97	1342.87 ± 493.5	0.6621
IL-12p70	24.74 ± 3.13	31.54 ± 4.73	0.2227
I-TAC	42.05 ± 4.06	107.24 ± 35.36	0.6299
MCP-2	49.07 ± 7.2	77.56 ± 26.86	0.5313
MCP-3	60.52 ± 13.14	264.09 ± 128.14	0.5382
MCP-4	406.6 ± 30.34	599.45 ± 121.46	0.6862
MMP-1	1933.57 ± 218.86	3412.24 ± 1048.31	0.9257
MMP-9	8957.7 ± 1631.17	9643.38 ± 1873.76	0.4984
PECAM-1	2286.03 ± 285.81	3207.37 ± 709.82	0.5686
TGFa	901.39 ± 329.31	1916.84 ± 556.24	0.6227
TGFb3	275.87 ± 61.11	2982.95 ± 1738.1	0.0395**
Tie-1	5181.73 ± 1903.04	20930.82 ± 7137.43	0.1115
Tie-2	357.31 ± 43.91	691.69 ± 189.69	0.4521
uPAR	3004.43 ± 268.21	4427.4 ± 858.59	0.6116
VEGF	790.81 ± 71.37	922.94 ± 221.57	0.3664
VEGF-R2	2219.33 ± 162.15	2535.33 ± 263.23	0.5271
VEGF-R3	536.32 ± 72.25	1265.24 ± 392.09	0.4368
VEGF-D	201.25 ± 145.92	1624.53 ± 671.27	0.0173**

* statistically significant value of < 0.05 for Student’s T-test

** statistically significant value of < 0.05 for Mann-Whitney Wilcoxon’s test

Patients with foetal DS had higher plasma concentrations of five angiogenic factors—TGFb1 (p = 0.039), angiostatin (p = 0.0142), I-309 (p = 0.0476), TGFb3 (p = 0.0395) and VEGF-D (p = 0.0173)—compared to patients with healthy foetuses (Table 2).

In Table 3 we have divided significant factors for pro or anti-agiogenic proteins.

**Table 3:** Significant proteins–pro/anti angiogenic function.

Significant protein	Function in angiogenic processes
TGFb1	proangiogenic
TGFb3	proangiogenic
I-309	proangiogenic
Angiostatin	antiangiogenic
VEGF-D	proangiogenic

## Discussion

Human chromosome 21 encodes the antiangiogenic factors as endostatin (ES), β-amyloid peptide (BAP) and Down Syndrome Critical Region 1 (DSCR-1) [18]. As in DS, there are three copies of the 21 chromosome; those genes are overexpressed. Therefore, individuals with DS were reported as a model for systemic antiangiogenic state due to overexpression of angiogenic factors. The novelty of our study is the increased expression of five angiogenic factors—TGFb1, TGFb3, angiostatin, VEGF-D, and I-309—found in maternal serum, encoded in the 19, 14, 6, X and 17 chromosomes, respectively.

Transforming growth factor beta 1 and 3 (TGFb1), (TGFb3) are two subtypes of the same protein, which are part of a vast cytokine group. These proangiogenic factors are natural modulators of cell transformation, proliferation and stimulation involved in the immunological and vascular system [19]. In the scientific literature, Hattori et al. have demonstrated a correlation between TGFb1 and DS. For example, Hattori et al. [20] and van der Wal et al. [21] found high concentrations of TGFb1 in plaques in Alzheimer's disease and DS pathologies. In addition, TGFb1 is a factor in responses to neurodegeneration: TGFb1 mRNA and protein were recently shown to increase in animal brains that cause local differentiation or neuronal death (which occurs in Alzheimer’s disease and DS) [22]. In our research, we found a statistically significant increase in the concentration of TGFb1 and TGFb3 in the plasma of women with foetal DS, which may confirm that these proteins are indeed involved in neurodegeneration processes in the foetus body. To our knowledge, there are no other studies that describe the level of TGFb proteins in the plasma of women with foetal DS. Furthermore, Dalgliesh et al. found increased levels of activin and inhibin in the plasma and placenta of women with foetal DS; these proteins belong to the same family as TGFb, ostensibly confirming our results [23].

Galambos et al. previously demonstrated the increased lung expression of antiangiogenic factors, including as COL18A1, COL4A3, TIMP3 and APP. Here, it was assumed that the increase of antiangiogenic factors’ expression leads to impaired lung vascular growth. Lung histology from patients with DS confirmed impaired lung vascular and alveolar development [18, 24]. In the present study, we found elevated levels of another antiangiogenic protein, angiostatin, in maternal plasma. Despite the fact that there are no other peer-reviewed studies about the levels of this protein in pregnant women with foetal DS, this protein is notable in the context of impaired angiogenesis and cardiovascular disease, which often occurs in individuals with DS [1]. Proteins such as metalloproteinases (MMPs), elastase and prostate-specific antigen (PSA), release angiostatin from plasminogen during enzymatic division [25]. Hopkins et al. observed reduced levels of plasminogen activator inhibitor-1 (PAI-I) in the blood of adults with DS, consequently resulting in increased plasminogen level activity [26]. The elevated levels and activity of plasminogen can be a potential source of angiostatin. In addition, Lambert-Messerlian et al. revealed increased concentrations of PSA in maternal serum from pregnancies affected by foetal DS [27]. As previously mentioned, PSA is one of the major activators of angiostatin synthesis.

Vascular endothelial growth factor D (VEGF-D) was also shown to be elevated in our participants. Generally, VEGF plays a major role in the growth of blood and lymphatic vessels. VEGF-D through enzymatic processes has conformation that fits and activates the VEGFR-2 and VEGFR-3 receptors [28]. Like the above-described proteins, VEGF-D has not been previously studied in DS, but VEGF has been tested in this disease model. Yao et al. [29] showed that VEGF selectively induces Down syndrome critical region 1 gene expression in endothelial cells, which results in the inhibition of angiogenesis and tumour growth [29]. Conversely, in our experiment, VEGF was not statistically significant. The mechanism and function of VEGF-D in foetal DS remains unclear, but this remains a notable protein requiring further research. The presence of three copies of chromosome 21 results in overexpression of its resident genes. Fuentes et al. presented the overexpression of the DSCR1, the product of the 21 chromosome gene, which acts as an inhibitor of calcineurin-mediated signalling pathways [30]. DSCR1 encodes a protein that inhibits VEGF-mediated angiogenic signalling by the calcineurin pathway, and its overexpression was confirmed by Baek et al. The diminution of calcineurin activity is reached by the overexpression of another 21 chromosome gene, Dyrk 1a. Scientists believe that DSCR1 and DYRK1A could be candidates for potential cancer therapy target [31].

I-309 (CCL1) is the last protein that was present at a higher concentration in the plasma of women with foetal DS. It is difficult to explain the elevated levels of I-309 in maternal plasma because of the small amount of research on processes involving this protein. Bernardini et al. Showed that I-309 is a new proangiogenic protein that connects to umbilical vein cells (HUVECs) and induces their chemotaxis, invasion, and differentiation [32]. Such a process could suggest that the protein is transplanted from the placenta into the mother's bloodstream.

In this publication, we found that selected angiogenic factors could be predictive factors for Down syndrome pregnancies and may play a role in the pathology of trisomy of chromosome 21. Currently, in the international literature, there is no relevant research focused on the roles of these factors in the pathogenesis of DS. Therefore, it is difficult to form definite conclusions about variations in the levels of angiogenic factors. However, due to the complexity of the pathomechanism responsible for DS, additional functional experiments should be performed.

## Supporting information

S1 File Raw data. (XLSX)
